# The prevalence of and factors related to reinfection with COVID‐19 in Ahvaz, Iran: A comparative cross‐sectional study

**DOI:** 10.1002/hsr2.1420

**Published:** 2023-07-21

**Authors:** Parvin Abedi, Poorandokht Afshari, Somayeh Ansari, Seyed Mohammad Alavi, Shohreh Dashtpayma, Homayon Amiri

**Affiliations:** ^1^ Department of Midwifery, Menopause Andropause Research Center Ahvaz Jundishapur University of Medical Sciences Ahvaz Iran; ^2^ Department of Midwifery, Reproductive Health Promotion Research Center Ahvaz Jundishapur University of Medical Sciences Ahvaz Iran; ^3^ Infectious and Tropical Diseases Research Center, Health Research Institute Ahvaz Jundishapur University of Medical Sciences Ahvaz Iran

**Keywords:** COVID‐19, prevalence, reinfection, vaccination

## Abstract

**Background and Aims:**

Reinfection with the coronavirus disease 2019 (COVID‐19) virus may be as serious as the first infection, exposing people to risks such as admission to hospital or even death. This study aimed to evaluate the prevalence of and the factors related to reinfection in Ahvaz, Iran.

**Methods:**

This was a comparative cross‐sectional study that was conducted on 200 reinfected individuals and 200 people who had once been infected with COVID‐19. Infection with COVID‐19 was confirmed using the polymerase chain reaction (PCR) test, and those with reinfection had to have a negative PCR test after recovery from the first infection and a positive PCR test for COVID‐19 > 90 days after the first infection. Data was collected using a questionnaire and a checklist. Data were analyzed using the Chi‐square test, independent *t*‐test, and logistic regression test.

**Results:**

Around 7000 reinfections were observed in this study, and the prevalence of reinfection was 0.59% in Ahvaz City. A large proportion of the participants in the control group, 133 (66.5%) received two doses of COVID‐19 vaccines compared with 110 (55%) in the reinfected group (*p* = 0.003), and 43 (21.5%) of reinfected participants did not receive any vaccine. Older people were 0.982 times more likely to get reinfected with COVID‐19 (95% confidence interval [CI]: 0.966–0.997). Also, those receiving vaccination once or twice were 2.311 and 2.498 times less likely to get reinfected with COVID‐19, respectively (95% CI: 1.093–4.887 and 1.281–4.872, respectively).

**Conclusion:**

The findings of this study showed that the prevalence of reinfection among people in Ahvaz City was 0.59%. Older individuals, those without vaccination or with suboptimal vaccination, and people with comorbidities were at a higher risk for reinfection. Health policymakers should pay more attention to factors related to reinfection with COVID‐19.

## 1 INTRODUCTION

The first cases of severe acute respiratory syndrome coronavirus 2 (SARS‐CoV‐2) were identified in Wuhan, China in December 2019, and soon after, the virus spread to other places in the world.^1^ In Iran, the first case of COVID‐19 infection was reported in February 2020 in Qom city.^2^ After thousands of deaths from the infection caused by SARS‐CoV‐2, the World Health Organization announced SARS‐CoV‐2 as a pandemic in 2020.^3^ According to the latest reports, 7,565,367 people have thus far been infected with COVID‐19 in Iran, of whom 144,779 have died.^4^ Developed countries, such as the United States started vaccination in less than one year after the start of the pandemic, while countries such as Iran resisted to import vaccines from other countries and hoped to defeat the disease with home‐grown vaccines.^5^

Reinfection with COVID‐19 virus may be as serious as the first infection. Vaccination played an important role in softening the impact of reinfection and the long‐term complications of the disease.^6^ A study in Iran showed that among 1492 individuals with confirmed COVID‐19 infection, the rate of reinfection was 0.33% in 1‐year follow‐up.^7^ In a retrospective study by Tavakoli et al.^8^ on 213,768 patients with confirmed the polymerase chain reaction (PCR) test, the rate of reinfection was 0.97%, and 66.6% of the reinfected participants had their second positive test 90 days after the first infection, with the higher chance for reinfection being observed among men, urban population, adolescents and health care providers. Rahman et al.^9^ found that individuals who get naturally infected were less likely to be reinfected compared to those who received vaccines. Sacco et al.^10^ also found that failure to receive vaccination and special variants such as Omicron were factors contributing to reinfection with COVID‐19. Wang et al.^11^ also found that reinfection with different variants of COVID‐19 is possible and that most reinfected people have similar symptoms as in the primary infection. Results of a systematic review including 19 studies and 1096 participants showed that the rate of reinfection was 0.65%, and reinfected individuals had the fewest symptoms.^12^

Despite previous studies conducted on reinfection of COVID‐19, this topic still seems to be a much‐needed line of inquiry, given the emergence of different variants of the virus. Therefore, this study was conducted to evaluate the prevalence and the factors related to reinfection in Ahvaz, Iran.

## 2 METHODS

This was a comparative cross‐sectional study conducted on 200 patients who were reinfected with COVID‐19 after 90 days from their primary infection, and 200 people who had one‐time infection. The Ethics Committee of Ahvaz Jundishapur University of Medical Sciences approved the design of the study (Ref. No: IR.AJUMS.REC.1400.245). All participants provided written informed consent before data collection.

### 2.1 Inclusion/exclusion criteria

Adult individuals who had been reinfected with COVID‐19 based on PCR test and from whose primary infection at least 90 days had passed based on the confirmation of an infectious disease specialist were recruited for this study. Furthermore, individuals with reinfection remained asymptomatic between the first and second infection and they had negative PCR tests. Individuals who had once been infected with the disease were recruited as the control group. Participants who claimed being reinfected but had no definite laboratory tests and those who were reinfected and then passed away were excluded from the study.

### 2.2 Sample size

The following formula was used for sample size:

$$n=\frac{\left(Z1-\frac{\alpha }{2}+\;Z1-\beta )(P1\left(1-P1\right)+P2(1-P2\right)}{\left(P1-P2\right)2}=190$$

$$Z1-\frac{\alpha }{2}=1.96$$

Confidence interval (CI): 95%

Z1−β=0.84,Power:80%

P1 = 325, P2 = 20% (using pilot study including 20 participants)

We reached a total number of 190, which increased to 200 participants for each group.

### 2.3 Procedure

The list of patients with COVID‐19 re‐infection was prepared from the Deputy of Health of Ahvaz Jundishapur University of Medical Sciences. Those who had two confirmed PCR tests with at least a 90‐day interval were selected. These individuals had a negative PCR test after recovery from their first infection, but then after at least 90 days, had a positive PCR test confirming their second infection.^13^

People who had been once infected were considered as a control group. Both groups received a phone call inviting them to attend a public health center to complete a questionnaire and a checklist.

### 2.4 Measures

A demographic questionnaire and a checklist were used to collect the data. The demographic questionnaire consisted of questions about age, the number of family members, sex, occupation, educational attainment, history of chronic disease, medication, and number of infections by COVID‐19. The checklist was used to record information about infection with COVID‐19. The content validity of the demographic questionnaire and the checklist were approved by 10 faculty members.

### 2.5 Variables

Independent variable: Reinfection with COVID‐19. Dependent variables: Prevalence and Related factors.

### 2.6 Statistics

Data analysis was done using IBM SPSS Statistics (version 23).^14^ Chi‐square test and independent *t*‐test (two sided) were used to compare categorical and continuous data, respectively. Logistic regression was used for testing the relationship between demographic characteristics and reinfection with COVID‐19. *p* < 0.05 was considered statistically significant.

## 3 RESULTS

**Figure 1:**
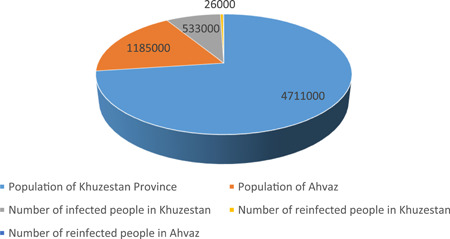
Number of infectetion and reinfection with coronavirus disease 2019 (COVID‐19) in Khuzestan province and Ahvaz.

This study was conducted in Ahvaz, the capital of Khuzestan province, southwest of Iran. The population of Khuzestan province is 4,711,000, and the population of Ahvaz is 1.185,000 according to the latest census.^15^ From the beginning of the COVID‐19 pandemic until September 2022, 533,000 individuals had been infected with COVID‐19 in the Khuzestan province, of whom 26,000 individuals were reinfected, which indicates a reinfection prevalence of 0.55%. Ahvaz accounts for around 7000 cases of reinfection, indicating a reinfection prevalence of 0.59% (Figure 1).

Table 1 shows the demographic characteristics of the participants. As evident from this table, individuals with reinfected with COVID‐19 were significantly older compared with the control group (*p* = 0.01). Most participants in the two groups were male, employees, and had a university degree. Significantly more individuals in the reinfection group had a history of chronic diseases such as diabetes, hypertension, and kidney disease (*p* = 0.002). Furthermore, patients with reinfection used more medication for their chronic diseases. Most of the participants in the reinfection group 162 (81%) had been infected with COVID‐19 twice.

**Table 1:** Demographic characteristics of participants in the reinfection and control groups.

Variables	Reinfected group *N* = 200	Control group *N* = 200	*p* Value
	Mean ± SD or *N* (%)		
Age (y)	36.59 ± 13.36	33.28 ± 14.23	0.01**
<25	51 (25.5)	88 (44)	0.002***
26–35	66 (33)	50 (25)	
36–45	38 (19)	22 (11)	
46–55	18 (9)	16 (8)	
>56	27 (13.5)	24 (12)	
The number of family	5.06 ± 2.3	5.2 ± 2.3	0.521**
Sex			
Male	121 (60.5)	106 (53)	0.158***
Female	79 (39.5)	94 (47)	
Occupation			
Unemployed	26 (13)	31 (15.5)	0.910***
Worker	66 (33)	62 (31)	
Employee	67 (33.5)	61 (30.5)	
Teacher	26 (13)	25 (12.5)	
Self‐employed	15 (7.5)	21 (10.5)	
Education			
High school	72 (36)	61 (30.5)	0.649***
High school diploma	32 (16)	42 (31)	
University education	96 (48)	97 (48.5)	
History of chronic disease			
None	63 (31.5)	74 (37)	0.002***
Diabetes	41 (20.5)	28 (14)	
Hypertension	34 (17)	17 (8.5)	
Diabetes and hypertension	10 (5)	15 (7.5)	
Heart disease	6 (3)	6 (3)	
Kidney disease	17 (8.5)	10 (5)	
Other*	29 (14.5)	50 (25)	
Medication			
Supplement	63 (31.5)	91 (45.5)	0.005***
Medication for chronic disease	108 (54)	73 (36.5)	
No medication	29 (14.5)	36 (18)	
Number of infection			
Once	0 (0)	200 (100)	
Twice	162 (81)	0 (0)	
Three times	29 (14.5)	0 (0)	
Four times	9 (4.5)	0 (0)	

* Including allergy with lung or eye symptoms,

** Independent *t*‐test,

*** Chi‐square test.

Table 2 shows the COVID‐19 infection routs and symptoms. As this table shows, a large proportion of the participants in the control group had received two doses of COVID‐19 vaccines, 133 (66.5%) compared with 110 (55%) in the reinfected group (*p* = 0.003). In total, 43 (21.5%) of the reinfected participants had not received any vaccine. The most common symptom in the reinfected participants and the control groups was fever/shivering (27.5% and 36.5%, respectively). The least common symptoms were gastrointestinal and body pain in the reinfected and control groups, respectively. The two groups were significantly different in terms of the symptoms of infection (*p* = 0.018). Persistent symptoms were cough/shortness of breath with a frequency of 40% and 29.5% in the reinfected and control groups, respectively.

**Table 2:** Routes and symptoms of infection in the reinfection and control groups.

Variables	Reinfected group first time *N* = 200	Reinfected group second time *N* = 200	Control group *N* = 200	*p* Value
	*N* (%)			
Family member who infected with COVID‐19				
Spouse	20 (10)		29 (14.5)	0.061*
Kids	17 (8.5)		26 (13)	
Parents	50 (25.5)		60 (30)	
Siblings	42 (21)		53 (13)	
None	71 (35.5)		32 (16)	
Vaccination				
One dose	47 (23.5)		48 (24)	0.003*
Two doses	110 (55)		133 (66.5)	
None	43 (21.5)		19 (9.5)	
Infection after vaccination				
Yes	21 (10.5)		0 (0)	
No	136 (68)		181 (90.5)	
Did not receive vaccine	43 (21.5)		19 (9.5)	
Symptoms of infection				
Sour throat	42 (21)	54 (27)	36 (18)	0.018*
Fever/shivering	55 (27.5)	66 (33)	73 (36.5)	
Cough/shortness of breath	30 (15)	39 (19.5)	26 (13)	
Gastrointestinal symptoms	4 (2)	21 (10.5)	0 (0)	
Myalgia/headache	15 (7.5)	4 (2)	3 (1.5)	
Anosmia and ageusia	18 (9)	7 (3.5)	10 (5)	
Fatigue	7 (3.5)	5 (2.5)	9 (4.5)	
Few symptoms together	29 (14.5)	4 (2)	43 (21.5)	
Persistent symptoms				
Cough/shortness of breath	80 (40)		59 (29.5)	
Myalgia/headache	4 (2)		5 (2.5)	
Anosmia and ageusia	24 (12)		12 (6)	
Fatigue	9 (4.5)		7 (3.5)	
Few symptoms together	7 (3.5)		5 (2.5)	
No symptom remained	76 (38)		112 (56)	

* Chi‐square test.

Table 3 shows the factors related to COVID‐19 infection in two groups of the reinfected and the control. Duration of the disease was significantly shorter in the second infection in the group of reinfection (*p* < 0.001). Admission to hospital and duration of hospitalization were significantly different between the two groups. Most participants claimed that they had experienced less severe symptoms in the reinfection period.

**Table 3:** Route of infection and suggested supplementary rules for severity of disease in reinfected groups and control group.

Variables	Reinfected group first time *N* = 200	Reinfected group second time *N* = 200	Control group *N* = 200	*p* Value
	Mean ± SD or *N* (%)		
Route of infection				
Family	33 (16.5)	26 (13)	45 (22.5)	0.337**
Workplace	33 (16.5)	29 (14.5)	37 (18.5)	
Participation in a party	32 (16)	10 (5)	27 (13.5)	
Not observing hygiene measures such as wearing a mask	19 (9.5)	28 (14)	23 (11.5)	
The did not know	83 (41.5)	107 (53.5)	68 (34)	
Duration of symptoms of the disease (days) mean ± SD	12.57 ± 9.72	9.89 ± 5.11	12.49 ± 6.29	<0.001*
	*N* (%)			
Admission to hospital				
Yes	34 (17)	17 (8.5)	43 (21.5)	0.310**
No	166 (83)	183 (91.5)	157 (78.5)	
Duration of hospitalization (days, mean ± SD**)**	6.45 ± 1.99	4.39 ± 1.21	6.3 ± 1.87	
Severity of disease in reinfection				
Worse	14 (7)		0 (0)	
Better	133 (66.5)			
Unchanged	53 (26.5)			

Abbreviation: SD, standard deviation.

* Independent *t*‐test.

** Chi‐square test.

The relationship of reinfection with some demographic factors was assessed using logistic regression, and the results are presented in Table 4. As evident from this table, older people were 0.982 times more likely to get reinfected with COVID‐19 (95% CI: 0.966–0.997). Also, those receiving vaccination once or twice were 2.311 and 2.498 times less likely to get re‐infected with COVID‐19 (95% CI: 1.093–4.887 and 1.281–4.872, respectively).

**Table 4:** Logistic regression for the association of demographic factors and factors related to the disease with reinfection.

Model	B	SE	Wald	*df*	Sig	Exp (B)	95% CI for Exp (B)	
Age	−0.019	0.008	5.331	1	0.021	0.982	0.966	0.997
Vaccination*								
Once	0.838	0.382	4.805	1	0.028	2.311	1.093	4.887
Twice	0.916	0.341	7.220	1	0.007	2.498	1.281	4.872

Abbreviations: CI, confidence interval; *df*, degrees of freedom; SE, standard error.

* Vaccination compared with no vaccination.

## 4 DISCUSSION

This study was designed to evaluate the prevalence of and the factors related to reinfection among COVID‐19 patients in Ahvaz, Iran. Like other coronaviruses, recovery from COVID‐19 does not provide permanent immunity, and there is always a possibility of reinfection.^16^ Therefore, studying the prevalence of reinfection and its associated factors is of paramount importance.

The results of the present study showed that the prevalence of reinfection in Ahvaz City was 0.59%. Tavakoli et al in a study on 213,768 individuals in Fars province in Iran, found that the prevalence of reinfection was 0.97% and that 14.9%, 18.5%, and 66.6% of their studied subjects had been reinfected 45, 89, and ≥90 days after their initial infection.^8^ The rate of reinfection in Tavakoli et al.'s^8^ study was more than what we found in the present study, which could be attributed to the fact that they recruited people in three intervals after the first infection we only recruited people if ≥90 days had passed from their first infection. In a systematic review including 19 studies and 1096 participants, Yinjun et al.^12^ found that the pooled reinfection rate was 0.65% (95% CI: 0.39–0.98), which is comparable to our findings. In another in another systematic review including 91 studies, Flacco et al.^17^ found a pooled rate of 0.97% for reinfection, and the risk of reinfection was statistically lower among vaccinated people and was significantly higher during the Omicron COVID‐19 wave. The reinfection rate in Flacco et al.'s study was again more than what we found in the present study, which could be explained by the fact that they studied countries such as the United States that had a higher reinfection rate (1.08, 95% CI: 0.93%–1.25%). According to Flacco et al., the rate of reinfection in Ahvaz is similar to that in European countries.

Our results showed that significantly more individuals in the reinfected group had a history of chronic diseases such as diabetes, hypertension, and kidney disease. Ren et al.,^18^ in a systematic review including 50 studies from 20 countries, found that 64 out of 118 patients with reinfection had comorbidities, the most common of which were hypertension and obesity, which confirms our findings.

Our results also showed that older people were 0.982 times more likely to get reinfected with COVID‐19. In line with this result, a study in Denmark showed that individuals younger than 60 have more than 80% protection against COVID‐19 for a year, but only 47% of people older than 60 years have a yearlong protection after the first infection.^19^

Our finding showed that the duration of the disease was significantly shorter in the second infection in the reinfection group. Wang et al.,^11^ in a case series study on 16 studies, found that 68.8% of the cases had similar severity in the second round of infection, and 18.8% had worse symptoms. Similar to our study, in a study in Serbia on 251,104 participants, of whom 13,792 were reinfected with COVID‐19, Medic et al.^20^ found that most of the reinfections had been recorded in January 2022, and most of the cases were mild, and death was very rare.

In the present study, 43 (21.5%) of the reinfected participants had not received any vaccine. Also, those who had received vaccination once or twice were 2.311 and 2.498 times less likely to get reinfected with COVID‐19. Levin et al.'s^21^ systematic review on 62 studies conducted in 25 developing countries found that the prevalence of COVID‐19 infection was almost two times higher in developing countries in comparison with developed countries. The higher rate of infection in developing countries could be attributed to poor access to vaccination and limited access to high‐quality health care.^21^

## 5 STRENGTHS AND LIMITATIONS OF THE STUDY

This is the first study to evaluate the rate of COVID‐19 reinfection and its related factors in Ahvaz City. Reinfection was confirmed by PCR test in this study. Despite its merits, this study has some limitations. First, Data about comorbidities were obtained based on the participant's self‐report, and we are not sure about their accuracy. Second, data on the COVID‐19 variant in the first infection or reinfection was not available.

## 6 CONCLUSION

The findings of the present study showed the prevalence of COVID‐19 reinfection among people in Ahvaz city was 0.59%. Individuals at an older age, receiving no or suboptimal vaccination, and/or having comorbidities were at a higher risk of reinfection. Health policymakers should pay more attention to factors related to reinfection with COVID‐19.

## AUTHOR CONTRIBUTIONS

**Parvin Abedi**: Conceptualization; formal analysis; investigation; methodology; software; supervision; validation; writing—original draft; writing—review & editing. **Poorandokht Afshari**: Conceptualization; formal analysis; funding acquisition; methodology; resources; supervision; writing—original draft; writing—review & editing. **Somayeh Ansari**: Conceptualization; methodology; software; writing—review & editing. **Seyed Mohammad Alavi**: Conceptualization; investigation; methodology; supervision; validation; writing—review & editing. **Shohreh Dashtpayma**: Conceptualization; data curation; investigation; methodology; validation; writing—review & editing. **Homayon Amiri**: Conceptualization; data curation; methodology; resources; software; writing—review & editing.

## CONFLICT OF INTEREST STATEMENT

The authors declare no conflict of interest.

## TRANSPARENCY STATEMENT

The lead author Poorandokht Afshari affirms that this manuscript is an honest, accurate, and transparent account of the study being reported; that no important aspects of the study have been omitted; and that any discrepancies from the study as planned (and, if relevant, registered) have been explained.

## Supporting information

Supporting information.

## Data Availability

DATA of this study will be available upon the reasonable request from the corresponding author. The data used in this manuscript will be available upon the request from corresponding author.
